# The Natural History and Diseases of the Human Teeth

**Published:** 1846-06

**Authors:** 


Art. XI.—*The Natural History and Diseases of the Human Teeth.* By Joseph Fox, M.R.C.S.L., Member of the Society of Medicine, Paris; Lecturer on the Structure and Diseases of the Teeth, at Guy's Hospital; and Surgeon Dentist to their Royal Highnesses the Dukes of Kent and Sussex. First American, from the third London edition. *Remodled, with an Introduction and numerous additions.* By Chapin A. Harris, M.D., D.D.S., Professor of Practical Dentistry and Dental Pathology in the Baltimore College of Dental Surgery; Fellow of the American Society of Dental Surgeons; Member of the Medico-Chirurgical Faculty of Maryland; Author of Principles and Practice of Dental Surgery, &c., &c. Illustrated with thirty plates. Philadelphia: Ed. Barrington & Geo. D. Haswell. 1846. Royal 8vo. pp. 440.

Within two years, we have been called upon to announce the appearance of three works on the Teeth, all of them voluminous, and two of them making a very imposing show. The one which forms the subject of the present notice, comes before the profession in our country with all the advantages of a finished mechanical execution. Its size, paper, type, and pictorial illustrations place it in the first class of American books relating to Dentistry. The American editor enjoys a high rank among dental surgeons, and he has brought his brethren under renewed obligations, by inducing the liberal publishers to send forth the celebrated work of Fox in so tasteful a style. Dr. Harris is determined that the dentists of the United States shall possess a library; and in his own excellent treatise, and the one which is now offered for their acceptance, he has placed within their reach the most valuable stores of information relative to the anatomy, physiology, and diseases of the teeth. Dentistry, both as an art and science, must advance in our country, under so judicious and industrious a culture.

